# Simultaneous Knockdown of Sprouty2 and PTEN Promotes Axon Elongation of Adult Sensory Neurons

**DOI:** 10.3389/fncel.2019.00583

**Published:** 2020-01-21

**Authors:** Sataporn Jamsuwan, Lars Klimaschewski, Barbara Hausott

**Affiliations:** Institute of Neuroanatomy, Medical University of Innsbruck, Innsbruck, Austria

**Keywords:** Sprouty2 (Spry2), PTEN (phosphatase and tensin homolog deleted on chromosome 10), DRG neurons, axon regeneration, pAkt (phosphorylated Akt)

## Abstract

Sprouty2 (Spry2) and phosphatase and tensin homolog deleted on chromosome 10 (PTEN) are both well-established regulators of receptor tyrosine kinase (RTK) signaling, and knockdown of Spry2 or PTEN enhances axon regeneration of dorsal root ganglia (DRG) neurons. The major role of Spry2 is the inhibition of the rat sarcoma RAS/extracellular signal-regulated kinase (ERK) pathway, whereas PTEN acts mainly as an inhibitor of the phosphoinositide 3-kinase (PI3K)/Akt pathway. In non-neuronal cells, Spry2 increases the expression and activity of PTEN, and PTEN enhances the amount of Spry2 by the inhibition of the microRNA-21 (miR-21) that downregulates Spry2. Applying dissociated DRG neuron cultures from wild-type (WT) or Spry2 deficient mice, we demonstrate that PTEN protein was reduced after 72 h during rapid axonal outgrowth on the laminin substrate. Furthermore, PTEN protein was decreased in DRG cultures obtained from homozygous Spry2−/− knockout mice. Vice versa, Spry2 protein was reduced by PTEN siRNA in WT and heterozygous Spry2+/− neurons. Knockdown of PTEN in DRG cultures obtained from homozygous Spry2−/− knockout mice promoted axon elongation without increasing axonal branching. Activation of Akt, but not ERK, was stronger in response to PTEN knockdown in homozygous Spry2−/− DRG neurons than in WT neurons. Together, our study confirms the important role of the signaling modulators Spry2 and PTEN in axon growth of adult DRG neurons. Both function as endogenous inhibitors of neuronal growth factor signaling and their simultaneous knockdown promotes axon elongation more efficiently than the single knockdown of each inhibitor. Furthermore, Spry2 and PTEN are reciprocally downregulated in adult DRG neuron cultures. Axon growth is influenced by multiple factors and our results demonstrate that the endogenous inhibitors of axon growth, Spry2 and PTEN, are co-regulated in adult DRG neuron cultures. Together, our data demonstrate that combined approaches may be more useful to improve nerve regeneration than targeting one single inhibitor of axon growth.

## Introduction

Peripheral nerves are provided with the ability to regenerate in response to injuries but recovery after peripheral nerve injury is still highly limited and functional outcomes are often poor (Skouras et al., 2011; Klimaschewski et al., 2013). Successful regeneration is dependent on extrinsic factors in the environment as well as on the intrinsic regenerative capabilities of neurons. One reason for the limitations in peripheral nerve regeneration is the presence of intracellular inhibitors of neuronal growth factor signaling (Duraikannu et al., 2019). Among them are Sprouty2 (Spry2) and the dual-specificity phosphatase and tensin homolog deleted on chromosome 10 (PTEN), which act as key regulators of receptor tyrosine kinase (RTK) signaling. The assigned major role of Spry2 is the inhibition of the rat sarcoma (RAS)/extracellular signal-regulated kinase (ERK) pathway (Mason et al., 2006; Hausott and Klimaschewski, 2019b), whereas PTEN has mainly been described as an inhibitor of the phosphoinositide 3-kinase (PI3K)/Akt pathway (Vazquez and Sellers, 2000; Krishnan et al., 2016). Both, the Ras/ERK and the PI3K/Akt pathway, are key players in axon growth of dorsal root ganglia (DRG) neurons (Chan et al., 2014; Hausott and Klimaschewski, 2019a).

Spry2 is highly expressed by adult DRG neurons and downregulation of Spry2 by shRNAs promotes axon growth of adult DRG neurons *in vitro* whereas overexpression of Spry2 inhibits axon growth (Hausott et al., 2009). DRG cultures from Spry2 knockout mice reveal enhanced axon elongation of heterozygous Spry2+/− neurons, whereas homozygous Spry2−/− neurons exhibit an axonal branching phenotype. *In vivo* studies with heterozygous Spry2+/− mice confirmed a better recovery following sciatic nerve crush and increased levels of GAP-43, a downstream target of ERK signaling (Marvaldi et al., 2015). Although Spry2 mRNA was not altered in response to a sciatic nerve lesion in our previous study, microRNA-21 (miR-21) is upregulated in the DRG after a peripheral nerve transection and reduces Spry2 protein levels in DRG cultures. Together, these studies confirm the important role of Spry2 in nerve regeneration (Hausott et al., 2009; Strickland et al., 2011).

PTEN is present in the intact and injured adult DRG with particularly high expression in small-diameter nociceptive neurons that bind isolectin B4 (IB4). Downregulation of PTEN increases axon growth of adult DRG neurons *in vitro* and this effect is even stronger in pre-lesioned neurons that were axotomized before the preparation of the culture. Furthermore, *in vivo* knockdown of PTEN promotes regeneration in response to a sciatic nerve transection. The effect of PTEN inhibition on axon growth of adult DRG neurons is independent of mammalian target of rapamycin (mTOR), whereas the same effect on axon growth of motor neurons is dependent on mTOR (Christie et al., 2010; Ning et al., 2010). PTEN is downregulated by miR-222 or by the ubiquitin ligase neural precursor cell expressed developmentally down-regulated protein 4 (NEDD4) in DRG neurons. MiR-222 is upregulated following sciatic nerve transection and promotes neurite outgrowth of adult DRG neurons, whereas knockdown of NEDD4 decreases axon growth of DRG neurons through upregulation of PTEN (Christie et al., 2012; Zhou et al., 2012). Although miR-21 downregulates PTEN in different cell types including neurons, it has no effect on PTEN protein levels in DRG neurons (Krichevsky and Gabriely, 2009; Strickland et al., 2011; Han et al., 2014).

Since individual downregulation of Spry2 or PTEN promotes axon regeneration and Spry2 interacts with PTEN in other cell types (Masoumi-Moghaddam et al., 2014), it was the aim of the current study to investigate the effects of simultaneous knockdown of Spry2 and PTEN on axon growth of adult DRG neurons *in vitro*. Knockdown of PTEN by siRNAs in DRG neurons obtained from homozygous Spry2−/− knockout mice promoted axon elongation and enhanced activation of Akt in comparison to each interference alone. Furthermore, Spry2 protein levels were decreased by PTEN knockdown and PTEN protein levels were lower in Spry2 deficient neurons. These findings demonstrate a beneficial effect of simultaneous knockdown of Spry2 and PTEN on axon growth and indicate an interrelated regulation of Spry2 and PTEN in DRG neurons.

## Materials and Methods

### Dissociated DRG Neuron Cultures and siRNA Transfection

Spry2-delta-ORF (Spry2^tm1.1Mrt^/Mmnc) knockout mice were obtained from MMRRC (Mutant Mouse Regional Resource Centre, University of North Carolina at Chapel Hill, Chapel Hill, NC, USA) and backcrossed on a 129 S1/SVImJ background as previously described (Marvaldi et al., 2015). DRG were harvested from 5 to 6 weeks old wild-type (WT), heterozygous Spry2+/− and homozygous Spry2−/− knockout mice. After removal of connective tissue, DRG were treated with Liberase™ DL research-grade (9 mg/100 ml in DMEM, Roche) two times for 30 min and with Trypsin-EDTA (Invitrogen) for 15 min. DRG were then washed and mechanically dissociated with a fire-polished Pasteur pipette in TNB 100 medium supplemented with protein-lipid complex (Biochrom), L-glutamine (Invitrogen), penicillin G sodium and streptomycin-sulfate (Invitrogen). Dissociated ganglia were then centrifuged at 500 rpm for 10 min through a 3.5% BSA gradient (Sigma Aldrich) to reduce the number of non-neuronal cells and debris. The pellet was washed in TNB 100 medium and centrifuged for 5 min at 760 rpm. Neurons were then resuspended and plated on glass coverslips or Nunc™ cell-culture treated multi dishes (24 well) pre-coated with 0.01% poly-L-lysine (Sigma Aldrich) and 10 μg/ml laminin (Sigma Aldrich). Neurons were cultivated in TNB 100 medium (Marvaldi et al., 2015) at 37°C with 10% CO_2_ according to the manufacturer’s instructions. PTEN knockdown was induced using the Accell siRNA delivery system (Dharmacon) designed to optimize transfection of primary cells. Transfection efficiency of Accell siRNA was assessed by imaging the uptake of Accell green non-targeting siRNA (D-001950-01). The target sequences of the Accell mouse PTEN SMARTpool siRNA mixture (siPTEN) were as follows: 5′-UGAUGAUGUAGUAAGGUUU-3′, 5′-GCGCUAUGUAUAUUAUUAU-3′, 5′-GUAGUAGGCUCAAAUAUAC-3′ and 5′-GUUACAAGUUACAUGUUUA-3′. Accell non-targeting siRNA pool (siNT; D-001910-10) was used as a control. After attachment of neurons after 2 h, they were transfected with 1 μM of Accell siRNA in Accell siRNA delivery media (Dharmacon) according to the manufacturer’s instructions (Ning et al., 2012). Neurons were used for experiments 24 h, 48 h or 72 h after transfection.

### Quantitative Real-Time PCR (qPCR)

To evaluate siRNA-induced knockdown of PTEN mRNA in DRG neuron cultures, RNA was collected 24 h, 48 h and 72 h after transfection using the ExCellenCT Lysis Kit (ABM) according to the manufacturer’s protocol. To determine endogenous PTEN mRNA in DRG tissue, lumbar DRG (L3-L5) were extracted, frozen immediately in liquid nitrogen and RNA was isolated using TRI Reagent RT (Molecular Research Center). RNA template was used for cDNA synthesis with iScript™ cDNA Synthesis Kit (Bio-Rad). Quantitative real-time PCR (qPCR) was performed with the CFX Connect™ Real-Time PCR Detection System (Bio-Rad) in a final volume of 20 μl with SsoAdvanced™ Universal SYBR^®^ Green Supermix (Bio-Rad) and QuantiTect^®^ primer assays (Qiagen) for PTEN (Mm_Pten_1_SG, QT00141568) and HPRT1 (Mm_Hprt_1_SG, QT00166768). The qPCR reactions were performed in 40 cycles with 15 s at 95°C and 30 s at 60°C.

### Immunocytochemistry and Axon Growth Analysis

DRG neuron cultures were fixed for 15 min with 4% buffered paraformaldehyde (PFA) at RT, permeabilized with 0.5% Triton X-100 for 15 min and blocked with 10% goat serum for 1 h. For axon growth analysis, neurons were pre-fixed with 1% PFA and 5% sucrose for 10 min before fixation to prevent the breakage of axons. Primary antibodies (Cell Signaling Technology, Danvers, MA, USA: anti-PTEN, #9188, 1:250; R&D systems, Minneapolis, MN, USA: anti-TuJ-1 neuron-specific β-III tubulin, MAB1195, 1:1,000) or isolectin GS-IB4 Alexa Fluor 488 (1:500, Invitrogen) diluted in 0.3% BSA was applied overnight at 4°C. After washing with PBS three times, secondary antibody (goat anti-rabbit Alexa Fluor 546, 1:1,000; Invitrogen) was added for 90 min at room temperature. No unspecific binding of secondary antibody was observed. Nuclei were stained with Hoechst (10 min, 1:20,000; Invitrogen) and coverslips were mounted in fluorescence mounting medium (Dako Omnis). Fluorescently labeled neuronal cell bodies and axons were imaged using an inverted fluorescence microscope (Zeiss AxioObserver Z1) equipped with a SPOT insight QE camera and VisiView^®^ imaging software. The fluorescence intensity of PTEN immunostaining of cell bodies was evaluated after background subtraction applying MetaMorph^®^ morphometry software. For axon growth analysis, the maximal distance of the longest axon, the number of branch points per neuron and the total axonal length were quantified using MetaMorph^®^ morphometry software. If necessary, axon images were stitched before analysis using Fiji (ImageJ win64) program. All morphologically intact neurons per coverslip with axons longer than two cell body diameters were analyzed. Branches that were longer than the cell body diameter were counted as branch points.

### Western Blotting

Total cell lysates from DRG cultures were prepared with RIPA II buffer supplemented with 20 μg/ml complete protease inhibitor cocktail (Roche Diagnostics) and phosphatase inhibitor cocktail II and III (1:100, Sigma Aldrich) followed by sonication. Protein concentrations were determined using Bradford protein assay reagent (Bio-Rad). Ten microgram of protein were separated by sodium dodecyl sulfate-polyacrylamide gel electrophoresis (SDS-PAGE) and transferred to Immobilon-FL-PVDF membrane (Millipore). Membranes were blocked with Odyssey^®^ blocking buffer (LI-COR Biosciences, Lincoln, NE, USA) in phosphate-buffered saline (PBS) and incubated with primary antibodies (Cell Signaling Technology, Danvers, MA, USA: anti-PTEN, #9188, 1:1,000; anti-pAkt, #4060, 1:2,000; anti-Akt, #2920, 1:2,000; anti-pERK, #9101, 1:1,000; anti-ERK, #9107, 1:2,000; anti-GAPDH, #5174, 1:1,000; Abcam: anti-Sprouty2, #60719, 1:500) overnight at 4°C. Secondary fluorescently labeled antibodies (LI-COR Biosciences, Lincoln, NE, USA: IRDye^®^ 680RD goat anti-mouse and IRDye^®^ 800CW goat anti-rabbit, 1:20,000) were detected by the Odyssey FC Imaging System (LI-COR Biosciences, Lincoln, NE, USA). The band intensities were quantified after background subtraction using Image Studio Lite Software version 5.2 (LI-COR Biosciences, Lincoln, NE, USA).

### Statistical Analysis

Data presented as bar graphs are mean values ± standard error of the mean (SEM) or standard deviation (SD) of at least three independent experiments. Statistical analysis was performed using one-way ANOVA followed by Bonferroni’s *post hoc* test. Differences with a *p* < 0.05 were considered statistically significant (*p < 0.05, **p < 0.01, ***p < 0.001 or ****p < 0.0001).

## Results

### Endogenous PTEN Levels Are Reduced in Culture

**Figure 1:**
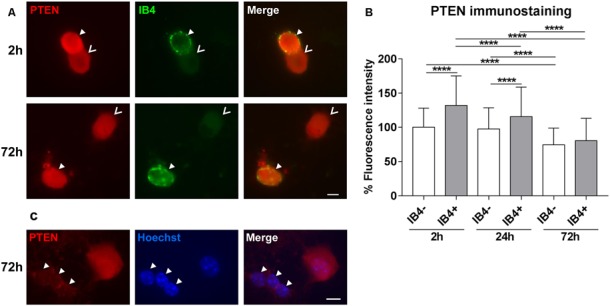
PTEN immunofluorescence in dorsal root ganglia (DRG) neuron subpopulations and glial cells. **(A)** Examples of PTEN immunoreactivity in IB4-positive (IB4+, filled arrowheads) and IB4-negative (IB4−, open arrowheads) DRG neurons. Bar = 10 μm. **(B)** PTEN fluorescence intensity was significantly higher in IB4-positive neurons after 2 h but this difference decreased in a time-dependent manner during cultivation. ****p < 0.0001 for the indicated comparisons, mean ± standard deviation (SD) of three independent experiments with a total number of neurons per group >180. **(C)** Immunoreactivity against PTEN is reduced in glial cells (arrowheads) compared to neurons. Bar = 10 μm.

In DRG tissue, PTEN is highly expressed by the lectin IB4-positive population of small neurons (Christie et al., 2010). Thus, we first investigated the distribution of PTEN in DRG subpopulations after 2 h, 24 h, and 72 h in culture. The PTEN immunoreactivity was significantly higher 2 h after plating in IB4-positive neurons reflecting the *in vivo* situation. However, immunofluorescence of PTEN decreased in a time-dependent manner in all DRG neuron populations during *in vitro* cultivation, and the difference between the IB4-positive and IB4-negative DRG subpopulation was strongly reduced after 72 h (Figures 1A,B). Glial cells present in DRG cultures were distinguished by their typical morphology of small nuclei that are surrounded by a narrow cytoplasmic margin. These associated glial cells revealed much weaker PTEN immunoreactivity as compared to DRG neurons (Figure 1C).

### PTEN Knockdown by Accell siRNA

**Figure 2:**
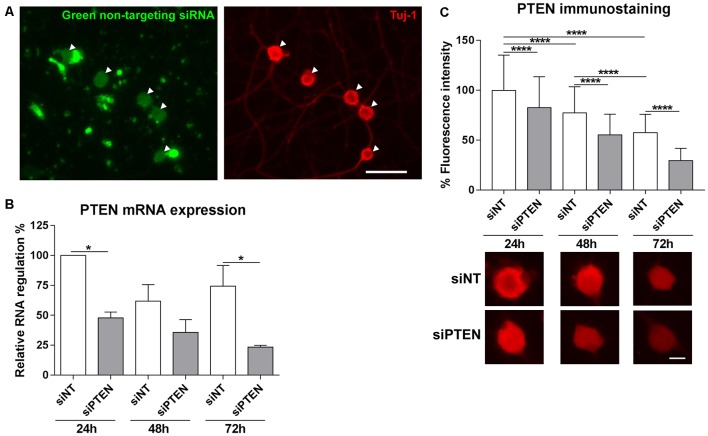
Transfection of adult DRG cultures with Accell siRNA and knockdown with PTEN siRNA (siPTEN) compared to non-targeting siRNA (siNT). **(A)** Uptake of Accell green non-targeting siRNA (arrowheads) into the cell bodies of dissociated DRG neuron cultures labeled with β-III tubulin (Tuj-1, red). Bar = 50 μm. **(B)** PTEN siRNA induces downregulation of PTEN mRNA in DRG neuron cultures as determined by quantitative real-time PCR (qPCR). Reduction of PTEN mRNA was observed after 24 h, 48 h and 72 h with the strongest downregulation compared to the non-targeting siRNA at 72 h. *p < 0.05 for the indicated comparisons, mean ± standard error of the mean (SEM) of three independent experiments. **(C)** Immunofluorescence measurements of PTEN following background correction indicate the reduction of endogenous PTEN protein levels in a time-dependent manner in neurons transfected with non-targeting siRNA, which is further reduced by PTEN siRNA after 24 h, 48 h and 72 h with the strongest downregulation at 72 h. ****p < 0.0001 for the indicated comparisons, mean ± SD of three independent experiments with a total number of neurons per group >400. Bar = 10 μm.

Methods to introduce RNA interference into adult DRG neurons are still challenging. We used the Accell siRNA technology from Dharmacon which consists of chemically modified siRNAs that are optimized for primary cells. First of all, the transfection efficiency of Accell siRNA was assessed by imaging the uptake of Accell green non-targeting siRNA into DRG neurons. Accell green control siRNAs were taken up by 95.0% (±11.0) of the neurons that were immunoreactive to β-III tubulin after 72 h in culture (Figure 2A). Next, we assessed the downregulation of PTEN by the Accell siRNA using qPCR. PTEN siRNA reduced mRNA expression after 24 h, 48 h and 72 h with the strongest downregulation at 72 h (by 68.4% compared to the non-targeting siRNA, Figure 2B). To confirm the effect of PTEN siRNA on protein levels, immunoreactivity against PTEN was measured after 24 h, 48 h and 72 h. PTEN siRNA reduced PTEN immunoreactivity in a time-dependent manner with the strongest reduction by almost 50% compared to non-targeting siRNA at 72 h (Figure 2C). Immunoreactivity against PTEN was again reduced in DRG neurons transfected with non-targeting siRNA at 48 h and 72 h in culture although the reduction in mRNA was not statistically significant (Figures 2B,C). Since the strongest mRNA and protein knockdown induced by the PTEN siRNA was observed after 72 h, further siRNA experiments were performed at this time point.

### Spry2 and PTEN Protein Levels Are Co-regulated in DRG Neurons

**Figure 3:**
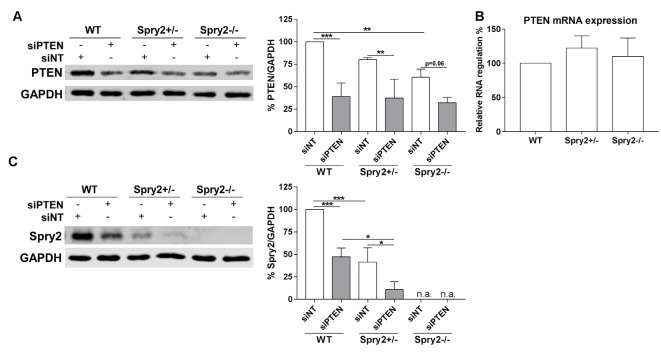
Western blot analysis of PTEN and Spry2 72 h after PTEN siRNA (siPTEN) transfection of wild-type (WT), heterozygous Spry2+/− and homozygous Spry2−/− DRG cultures compared to non-targeting siRNA (siNT). **(A)** PTEN protein decreased in response to PTEN siRNA in WT, heterozygous Spry2+/− and homozygous Spry2−/− DRG cultures. In addition, PTEN was reduced in homozygous Spry2−/− cultures transfected with non-targeting siRNA as compared to WT cultures. **p < 0.01, ***p < 0.001 for the indicated comparisons, mean ± SD of three independent experiments. **(B)** No difference in PTEN mRNA expression was observed in DRG tissue from heterozygous Spry2+/− and homozygous Spry2−/− knockout mice compared to the WT. Mean ± SEM of four independent experiments. **(C)** Spry2 protein decreased in heterozygous Spry2+/− cultures and was absent in homozygous Spry2−/− cultures (n.a., not applicable). In response to PTEN siRNA, Spry2 was markedly reduced in WT and heterozygous Spry2+/− cultures. *p < 0.05, ***p < 0.001 for the indicated comparisons, mean ± SD of three independent experiments.

Knockdown of PTEN protein by siRNA was also confirmed by Western blotting after 72 h (Figure 3A). Although PTEN and Spry2 are expressed by adult DRG neurons *in vitro* and *in vivo* (Hausott et al., 2009; Christie et al., 2010; Marvaldi et al., 2015), a possible correlation between Spry2 and PTEN in DRG neurons has not been investigated so far. PTEN protein was significantly reduced in homozygous Spry2−/− knockout cultures compared to the WT although no reduction in PTEN mRNA levels was observed in DRG tissue suggesting a possible post-transcriptional regulation of PTEN in response to Spry2 knockdown (Figures 3A,B). DRG cultures from heterozygous Spry2+/− mice revealed reduced Spry2 protein levels and complete knockdown of Spry2 protein was observed in homozygous Spry2−/− DRG cultures. Furthermore, PTEN siRNA markedly reduced Spry2 protein levels of WT and heterozygous Spry2+/− DRG cultures (Figure 3C). These findings imply the reciprocal regulation of Spry2 and PTEN in adult DRG neuron cultures.

### Simultaneous Knockdown of Spry2 and PTEN Promotes Axon Elongation

**Figure 4:**
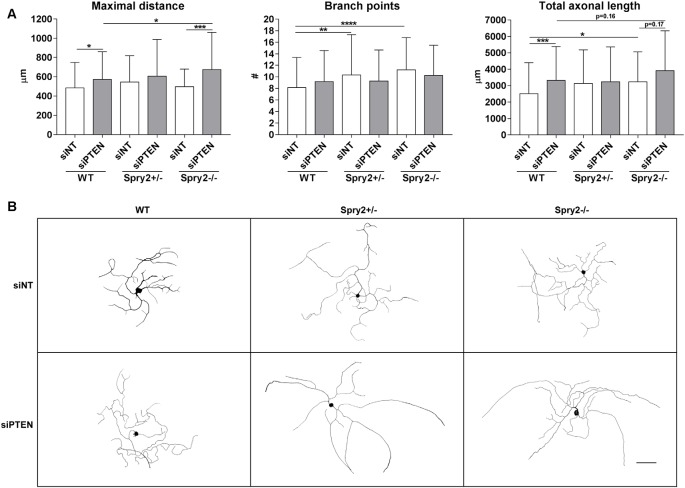
Axon growth 72 h after PTEN siRNA (siPTEN) transfection of WT, heterozygous Spry2+/− and homozygous Spry2−/− DRG cultures compared to non-targeting siRNA (siNT). **(A)** PTEN downregulation increased the total axonal length and the length of the longest axon (maximal distance) of WT neurons without enhancement of axonal branching. Heterozygous Spry2+/− and homozygous Spry2−/− DRG cultures revealed increased branching after 72 h. In homozygous Spry2−/− DRG neurons PTEN siRNA increased the maximal distance. *p < 0.05, **p < 0.01, ***p < 0.001, ****p < 0.0001 for the indicated comparisons, mean ± SD of three independent experiments with a total number of neurons per group >90. **(B)** Representative examples of neuronal morphologies of WT, heterozygous Spry2+/− and homozygous Spry2−/− DRG cultures 72 h after transfection with non-targeting or PTEN siRNA. Bar = 100 μm.

We observed the effects of Spry2 knockdown on axon outgrowth of dissociated DRG neurons after 24 h in our previous study with improved axonal elongation of heterozygous Spry2+/− cultures and increased axonal branching of homozygous Spry2−/− cultures (Marvaldi et al., 2015). Since optimal knockdown with Accell siRNA is usually observed after 72 h (according to the manufacturer’s instructions) and PTEN knockdown was stronger after 72 h than after 24 h in our cultures, axon growth was analyzed after 72 h. After 72 h of PTEN knockdown, the maximal distance of the longest axon (by 16.0%) and the total axonal length (by 32.3%) increased in WT neurons, whereas the number of branch points did not significantly change (Figures 4A,B). Neurons from heterozygous Spry2+/− and homozygous Spry2−/− knockout mice revealed both a branching phenotype at 72 h, while the maximal distance was not increased. However, knockdown of PTEN promoted axonal elongation by homozygous Spry2−/− deficient DRG neurons significantly stronger (by 36.0%) than PTEN single downregulation without increasing the number of branch points. Together, simultaneous knockdown of Spry2 and PTEN promoted axon elongation stronger than PTEN single knockdown without further enhancing axon branching that is observed after a single knockdown of Spry2 after 72 h.

### Simultaneous Knockdown of Spry2 and PTEN Enhances Akt Activation

**Figure 5:**
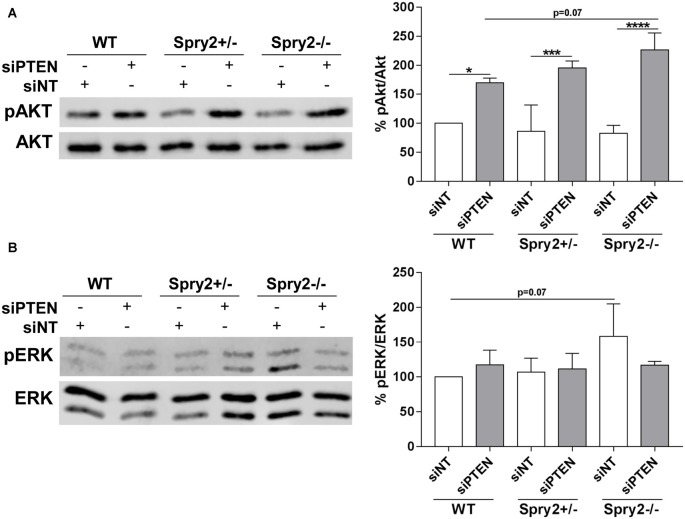
Western blot analysis of Akt and ERK phosphorylation 72 h after PTEN siRNA (siPTEN) transfection of WT, heterozygous Spry2+/− and homozygous Spry2−/− DRG cultures compared to non-targeting siRNA (siNT). **(A)** pAkt/Akt ratios were enhanced in response to PTEN downregulation and this effect was stronger in homozygous Spry2−/− DRG cultures than in WT cultures. *p < 0.05, ***p < 0.001, ****p < 0.0001 for the indicated comparisons, mean ± SD of three independent experiments. **(B)** pERK/ERK ratios were increased in homozygous Spry2−/− DRG cultures but not in response to PTEN downregulation. Mean ± SD of three independent experiments.

PTEN is a well-established inhibitor of Akt signaling and the described main function of Spry2 is the inhibition of the ERK pathway (Vazquez and Sellers, 2000; Mason et al., 2006). Therefore, phosphorylation of Akt and ERK in response to the simultaneous knockdown of Spry2 and PTEN in DRG cultures was analyzed by Western blotting. As demonstrated in our previous studies (Hausott et al., 2009; Marvaldi et al., 2015), immunoblotting revealed no changes in Akt phosphorylation in Spry2 deficient DRG neurons (Figure 5A). Thus, the modest reduction of PTEN protein observed in Spry2 deficient DRG neurons (Figure 3A) did not induce Akt activation. However, PTEN knockdown promoted Akt phosphorylation as expected and this effect was stronger in homozygous Spry2−/− DRG neurons than in WT neurons (*p* = 0.07). No difference in phosphorylation of ERK was observed in response to PTEN knockdown. In homozygous Spry2−/− knockout cultures, activation of ERK increased with a *p*-value of 0.07 but this effect was abolished by the PTEN siRNA (Figure 5B).

## Discussion

It was demonstrated before by our group and others that single knockdown of Spry2 or PTEN promotes axon growth of adult DRG neuron cultures (Hausott et al., 2009; Christie et al., 2010; Marvaldi et al., 2015). In this study, we provide for the first time evidence that simultaneous knockdown of Spry2 and PTEN has a stronger effect on axon elongation and activation of Akt than downregulation of PTEN alone. Furthermore, we demonstrate that PTEN protein levels decrease during *in vitro* cultivation and that Spry2 and PTEN protein levels are downregulated reciprocally in adult DRG neuron cultures.

PTEN is highly expressed by the lectin IB4-positive population of small DRG neurons 3 days after sciatic nerve lesion (Christie et al., 2010). In DRG neuron culture, PTEN immunoreactivity was significantly higher in IB4-positive neurons 2 h after plating reflecting the *in vivo* situation after lesion. During cultivation PTEN immunofluorescence decreased and after 72 h IB4-positive and IB4-negative DRG subpopulations revealed similar levels of PTEN. The reduction of PTEN during cultivation may facilitate neurite outgrowth especially of IB4-positive neurons after 72 h, which are limited in their regenerative capacity *in vitro* after 24 h (Leclere et al., 2007). PTEN is expressed by Schwann cells within the sciatic nerve and in regenerating axons but PTEN levels in glia appear weaker than in regenerating axons (Christie et al., 2010). Glial cells present in our cultures exhibited reduced immunoreactivity as compared to DRG neurons. Together, these studies indicate that PTEN expression in cell bodies and axons of DRG neurons is higher than in glial cells and that PTEN levels decrease in neurons during the process of neurite outgrowth.

PTEN protein is reduced in homozygous Spry2−/− DRG cultures and Spry2 is reduced in WT and heterozygous Spry2+/− DRG cultures by PTEN knockdown. Spry2 and PTEN are often concurrently deregulated in tumors and miRNAs or ubiquitin ligases appear to be involved in these processes (Wang et al., 2007; Patel et al., 2013; Zhang et al., 2016). PTEN negatively regulates miR-21 expression *via* the RNA-regulatory protein ribonuclease/angiogenin inhibitor 1 (RNH1), thereby preventing the downregulation of Spry2 by miR-21 in glioma cells (Kim et al., 2011; Kwak et al., 2011). MiR-21 is upregulated in response to a peripheral nerve lesion and downregulates Spry2, but not PTEN, in DRG neurons (Strickland et al., 2011). During the preparation of DRG cultures, neurons are axotomized as well, which may upregulate miR-21 in DRG cultures since PTEN is downregulated in cultures as observed in our study. In tumor cells and primary embryonic fibroblasts, Spry2 increases the amount of PTEN and decreases its phosphorylation, thereby enhancing the activity of PTEN to suppress phosphorylation of Akt (Edwin et al., 2006; Feng et al., 2011). Vice versa, Spry2 knockdown enhances phosphorylation and nuclear translocation of PTEN (Patel et al., 2013). PTEN is downregulated by the miR-222, which itself increases following sciatic nerve transection and promotes neurite outgrowth by downregulation of PTEN (Zhou et al., 2012). The ubiquitin ligase NEDD4 downregulates Spry2 and PTEN, and knockdown of NEDD4 inhibits axon growth of DRG neurons (Edwin et al., 2010; Christie et al., 2012). PTEN mRNA levels were not reduced in homozygous Spry2−/− DRG tissue from uninjured animals although PTEN protein was reduced in homozygous Spry2−/− DRG cultures suggesting a possible post-transcriptional regulation of PTEN in response to Spry2 knockdown. However, cultured DRG neurons undergo a lesion process which may also change gene expression. Thus, the exact mechanisms of the reciprocal downregulation of Spry2 and PTEN protein, as well as downregulation of the latter during DRG cultivation, remain to be elucidated in future studies.

Simultaneous knockdown of Spry2 and PTEN had a more beneficial effect on elongating axon growth of adult DRG neurons than single knockdown of PTEN. We used the Accell PTEN siRNA optimized for primary cells which revealed the strongest knockdown at 72 h in culture. Furthermore, the effect of PTEN downregulation on axon growth of WT neurons was only visible after 72 h but not after 24 h (data not shown). Other studies using Accell siRNAs in neurons did neither observe sufficient protein knockdown nor stimulation of axon growth before 72 h (Ning et al., 2012; Hannila et al., 2013). In contrast to our previous study with heterozygous Spry2+/− and homozygous Spry2−/− DRG cultures which was performed after 24 h (Marvaldi et al., 2015), both heterozygous Spry2+/− and homozygous Spry2−/− neurons exhibited a branching phenotype after 72 h. The PTEN siRNA did not further increase axonal branching of heterozygous Spry2+/− and homozygous Spry2−/− neurons but elongative axon growth was concurrently enhanced in homozygous Spry2−/− knockout cultures. Thus, knockdown of PTEN transformed the branching phenotype of homozygous Spry2−/− knockout cultures into a more elongative phenotype, which is important for successful nerve regeneration particularly at the lesion site. Other studies demonstrated as well that the combined deletion of different inhibitory signaling molecules has a better effect on axon growth than single inhibition only. Simultaneous knockdown of PTEN and suppressor of cytokine signaling 3 (SOCS3), an inhibitor of the Janus kinase/signal transducer and activator of transcription (JAK/STAT) pathway, reveals a better effect on regeneration after sciatic or optic nerve crush lesion (Sun et al., 2011; Gallaher and Steward, 2018). Although co-deletion of PTEN and SOCS3 increases sciatic nerve regrowth after lesion to a similar extent as PTEN deletion alone, simultaneous knockdown of PTEN and SOCS3 leads to more rapid recovery of thermo- and mechanosensation (Gallaher and Steward, 2018). By contrast, other studies observed that enhanced axon regrowth after lesion in response to robust mTOR activation inhibits proper innervation of the epidermis due to excessive branching (Abe et al., 2010). Our *in vitro* results indicate that the simultaneous knockdown of Spry2 and PTEN promotes elongative axon growth which is a precondition for successful regeneration. Furthermore, the combination of PTEN knockdown and activation of rapidly accelerated fibrosarcoma (RAF) or cyclic adenosine monophosphate (cAMP) enhances optic nerve regeneration (de Lima et al., 2012; Zhong, 2015). It is well known that axon growth is influenced by multiple inhibitory and growth-promoting factors especially in the central nervous system. Our results confirm that the development of combined approaches may be useful to improve nerve regeneration in the peripheral nervous system as well. Since the *in vivo* situation is much more complex in many respects, the effect of simultaneous knockdown of Spry2 and PTEN on sciatic nerve regeneration needs to be investigated in future studies.

Activation of Akt did not change in heterozygous Spry2+/− and homozygous Spry2−/− neurons confirming our previous studies (Hausott et al., 2009; Marvaldi et al., 2015). Although PTEN protein was reduced in homozygous Spry2−/− cultures, pAkt was unchanged indicating that the modest reduction of PTEN by Spry2 knockdown is not sufficient to activate detectable amounts of Akt. In response to the stronger PTEN reduction induced by the siRNA, activation of Akt was observed and this activation was stronger in homozygous Spry2−/− DRG neurons than in WT neurons, although knockdown of Spry2 alone had no effect on Akt activation. It was demonstrated before that simultaneous knockdown of Spry2 and PTEN results in higher pAkt activation than single knockdown of PTEN in prostate cancer tissue (Gao et al., 2012). The Akt pathway plays a major role in adult axon outgrowth and it is known that activation of Akt increases axonal branching in developing and adult DRG neurons (Markus et al., 2002; Jones et al., 2003). Surprisingly, our study revealed enhanced activation of pAkt by simultaneous knockdown of Spry2 and PTEN that resulted in improved axonal elongation without further increasing the branching phenotype induced by single knockdown of Spry2 after 72 h. Furthermore, single knockdown of PTEN in WT cultures enhanced activation of Akt and axon elongation but not axonal branching after 72 h. Activation of ERK was enhanced in homozygous Spry2−/− neurons after 72 h as observed in our previous studies after 24 h (Marvaldi et al., 2015). However, knockdown of PTEN, which strongly activated Akt, diminished this effect on ERK activation. It has been shown before that strong activation of Akt inhibits the ERK pathway by serine phosphorylation of RAF or forkhead box protein O1 (FOXO1; Zimmermann and Moelling, 1999; Lee et al., 2008; Pan et al., 2017).

Taken together, our study confirms the important role of Spry2 and PTEN in axon growth of adult DRG neurons. Both are endogenous inhibitors of neuronal growth factor signaling and their simultaneous knockdown promotes axon elongation stronger than the single knockdown of each inhibitor. *In vivo* studies confirm the significance of the endogenous inhibitors Spry2 and PTEN that are downregulated in response to a sciatic nerve lesion by miR-21 or miR-222, respectively. Axon growth is influenced by multiple factors and our results confirm that combined approaches targeting different inhibitors of axon growth may be useful to improve peripheral nerve regeneration.

## Data Availability Statement

All datasets generated for this study are included in the article.

## Ethics Statement

Ethical review and approval was not required for the animal study because the tissue was collected postmortem. Animal breeding was performed under the permission of the Austrian government: BMWFW-66.011/0120-WF/V/3b/2016.

## Author Contributions

BH: conceptualization, writing—original draft preparation and funding acquisition. BH and SJ: methodology and formal analysis. BH, SJ, and LK: writing—review and editing.

## Conflict of Interest

The authors declare that the research was conducted in the absence of any commercial or financial relationships that could be construed as a potential conflict of interest.
